# The Fire at the College of Physicians and Surgeons, Chicago

**Published:** 1901-09

**Authors:** Frank B. Earle

**Affiliations:** Secretary. Chicago, Ill.


*To the Editor Buffalo Medical Journal :*

### The Fire at the College of Physicians and Surgeons, Chicago.

*To the Editor Buffalo Medical Journal :*

Undoubtedly you have noticed through press reports the damage our old college building suffered through fire the 25th of June. A great many incorrect reports are in circulation regarding the continuance of our work, facilities, and the like. Kindly inform your readers that the administration of the college has suffered no serious disturbance in consequence of the fire and that we are now better housed and equipped than ever before and running quite as smoothly.

FRANK B. EARLE, Secretary.

CHICAGO, Ill., August 2, 1901.

